# Automatic early detection of pathological signs following primary total hip arthroplasty using radiographs, clinical scores, and comorbidities

**DOI:** 10.1371/journal.pone.0348790

**Published:** 2026-06-15

**Authors:** Sarah Galante, Anna Corti, Riccardo Stuani, Katia Chiappetta, Mattia Loppini, Valentina D. A. Corino

**Affiliations:** 1 Department of Electronics, Information and Bioengineering, Politecnico di Milano, Milan, Italy; 2 Department of Biomedical Sciences, Humanitas University, Pieve Emanuele, Milan, Italy; 3 IRCCS Humanitas Research Hospital, Rozzano, Milan, Italy; 4 Cardio Tech-Lab, Centro Cardiologico Monzino IRCCS, Milan, Italy; University of Alabama at Birmingham, UNITED STATES OF AMERICA

## Abstract

The growing prevalence of total hip arthroplasty (THA) revisions, along with their generally poorer outcomes compared to primary procedures, emphasizes the urgent need for early detection of primary THA failure. This study proposes a model that integrates radiographic, clinical, and comorbidity data to automatically detect pathological signs within one year after primary THA. The dataset included two independent patient cohorts: the first comprised 400 patients, leading to 801 radiographs with pathological signs and 785 without; the second included 155 patients, resulting in 417 radiographs with pathological signs and 508 without. After preprocessing, the dataset was split into training, validation, and test sets. Three models were developed, one for each data type. A deep learning framework was applied to the radiographic data, while multiple machine learning classifiers were trained on the clinical and comorbidity data. Predictive probabilities were obtained for each subset and data type, and the final combined model was generated by averaging the predicted probabilities from all individual models. The final combined model achieved an F1 score of 0.72 (95% CI: 0.65–0.79), a balanced accuracy of 0.69 (95% CI: 0.63, 0.77), and an area under the curve (AUC) of 0.72 (95% CI: 0.65, 0.79) on the internal test set. On the external validation set, it achieved an F1 score of 0.66 (95% CI: 0.62, 0.70), a balanced accuracy of 0.62 (95% CI: 0.59, 0.66), and an AUC of 0.67 (95% CI: 0.62, 0.72). The results demonstrate the potential of the developed approach to automatically detect early pathological signs of THA, enabling virtual follow-up and potentially reducing the burden on clinicians.

## Introduction

Total hip arthroplasty (THA) is widely recognized as the gold standard surgical intervention for the treatment of various hip conditions including end-stage osteoarthritis [1,2]. With the increase of life expectancy and an aging population, combined with improved access to surgical care, the demand for THA has significantly increased [3]. Over 1 million THAs are currently performed in the US every year, and this number is expected to rise of around 129% by 2030 [4]. Although more common among the elderly, approximately 25% of joint replacement recipients are under the age of 65 [5], and this proportion is expected to rise in the coming decades [6]. This younger demographic is particularly relevant, as THA is associated with a cumulative revision rate of up to 13% at 10 years, with younger patients facing a higher risk of failure [7]. Revision surgeries might be required due to various failure causes, such as aseptic loosening, infection, instability, persistent pain, bearing surface wear and osteolysis [8,9]. Given the growing number of THA procedures, the burden of revision THA is also expected to rise significantly with projections indicating an increase of 43% to 70% between 2014 and 2030 [10,11]. Therefore, it is important to early identify potential complications and failures by consistently conducting radiographic follow-ups after primary THA. This approach can help reduce healthcare costs, improve the likelihood of managing complications effectively, and enable the implementation of proactive preventive strategies. However, the increase of THA procedures results in an increasing number of radiographic examinations to be analyzed, thereby challenging clinicians’ capacity to allocate sufficient time for comprehensive and detailed evaluations.

To address this challenge, artificial intelligence (AI) tools are well suited, as they can rapidly process extensive datasets and thus represent a valuable resource to reduce the burden on clinicians. Recent studies developed machine learning (ML) and deep learning (DL) models that leverage either clinical, demographic, radiographic datasets or surgical variables for the prediction of THA failure [5,12–20]. Interestingly, Shah et al. [12] developed a DL model based on preoperative radiographs and clinical data, including patient history and comorbidities. Their study demonstrated that incorporating clinical data substantially enhanced the model’s ability to detect implant loosening before revision surgery [12]. However, to date, most models primarily rely on preoperative radiographs obtained before revision surgery to make their predictions [12–15].

To the best of our knowledge, no previous studies have combined imaging, clinical, and demographic data to predict potential pathological features using radiographs acquired just a few months after primary THA. This approach offers a more proactive assessment compared to models relying on preoperative images obtained immediately before revision surgery.

Therefore, the aim of this study is to develop a combined DL and ML pipeline, in which DL is used to analyze imaging data and ML integrates these with clinical and demographic data for the early detection of pathological signs following primary THA. In this study, pathological signs are defined as early indicators of potential complications, which do not correspond to confirmed prosthesis failure. Specifically, in addition to the x-rays available for each patient, clinical data from the Harris Hip Score (HHS) questionnaire, along with demographic information and comorbidities, were incorporated to improve classification performance. While radiographic data offer information on the periprosthetic condition, incorporating additional factors such as pain, body weight, and comorbidities can provide valuable complementary insights for identifying potential pathological signs.

## Methods

### Population

This study involved two independent cohorts of patients who underwent primary THA. The first cohort, retrospectively enrolled, (cohort A) was used for model development and internal testing, while the second cohort, prospectively collected, (cohort B) served for external validation of the model. The study was approved by the Institutional Ethical Committee (protocol code 272/23 approved on the 14/11/2023), Italy, and all patients gave their written informed consent. Access to the data may be requested by contacting cetlombardia5@humanitas.it. the code used for the analyses is publicly available in a GitHub repository and archived in Zenodo at https://doi.org/10.5281/zenodo.19064049.

The cohort A included 400 patients who had undergone primary THA, and whose clinical, demographic, and radiographic data were retrospectively collected from the Arthroplasty Register of Fondazione Livio Sciutto. The retrospective recruitment phase started on February 5, 2020, and ended on January 11, 2023, and the data were accessed for research purposes on 01/02/2025. All patients were identified using anonymized study codes that prevented any personal identification after data collection. For each patient, at least two clinical and radiographic evaluations performed within the first 12 months after surgery were available, including one follow-up at 1 month and a second follow-up at either 3, 6, or 12 months postoperatively, resulting in a total of 800 available follow-ups. Both asymptomatic and symptomatic patients were considered, with radiographs showing either normal features or pathological findings associated with the joint prosthesis and periprosthetic bone, such as implant malalignment, hypertrophy or atrophy of the periprosthetic bone, radiolucency lines, osteolysis, or prosthetic loosening.

For each follow-up, at least one radiographic projection was available, either an anteroposterior (AP) or a lateral (LAT) view, and in most cases both. In particular, from 400 patients, 1586 radiographs were available. Each follow-up was also associated with clinical data from HHS questionnaire, including total score, pain assessment, and range of motion, along with demographic information (height, weight) and relevant comorbidities (Table 1). Importantly, in this study each follow-up was treated as an independent observation, and the pathological label was assigned at follow-up level rather than at the patient one. Therefore, the clinical and demographic characteristics shown in Table 1 are expressed per follow-up, reflecting the total number of observations instead of the number of patients.

**Table 1:** Clinical and demographic characteristics of cohort A.

	Total	Pathological	Non-pathological
Follow-up number	798	401	397
Height (cm)	169.17 ± 9.71	170.08 ± 9.79	168.24 ± 9.57
Weight (Kg)	75.54 ± 16.04	75.57 ± 15.16	74.02 ± 16.86
HHS score (0–100)	93.4 ± 9.7	95.1 ± 8.7	91.7 ± 10.4
Hypertension	270 (33.75%)	122 (15.25%)	148 (18.5%)
Cardiac diseases	110 (13.75%)	48 (6%)	62 (7.75%)
Liver diseases	90 (11.25%)	49 (6.13%)	41 (5.13%)
Thyroid diseases	96 (12%)	49 (6.13%)	47 (5.88%)

Characteristics are expressed per follow-up, as each follow-up represents an independent data point. Height, weight and HHS score were reported in terms of mean ± standard deviation, and the other characteristics were reported in terms of percentage.

Among these follow-ups, 401 presented pathological features on radiographs indicative of potential implant failure, thus constituting the *pathological group*, corresponding to 801 radiographs, while the remaining 397 follow-ups, without such findings, were classified as the *non-pathological group* corresponding to 785 radiographs. One patient was excluded from the analysis due to missing HHS data, leading to a total of 798 available follow-ups for model development.

The cohort B consisted of 155 patients who were prospectively enrolled and monitored according to a predefined post-THA follow-up schedule, with clinical and radiographic evaluations at 1 month (±10 days), 3 months (±15 days), 6 months (±1 month), and 12 months (±2 months) after primary THA surgery. Depending on the orthopedic surgeon’s assessment of the clinical and radiographic findings, the follow-up schedule could be modified.

The prospective enrollment period extended from November 14, 2023, to July 24, 2024. Data were accessed for research purposes on 01/04/2025, only after completion of the clinical follow-up, and all records were anonymized prior to analysis. At no stage did the investigators have access to information that could identify individual participants.

For each patient, demographic and comorbidity information, as well as clinical data were collected similarly to cohort A. Across cohort B, 206 follow-ups exhibited pathological findings, corresponding to 417 images, while 256 follow-ups without pathological findings corresponded to 508 images.

This cohort was used for external validation of the predictive model, thereby enhancing the robustness and generalizability of its performance.

### Image preprocessing

**Fig 1:**
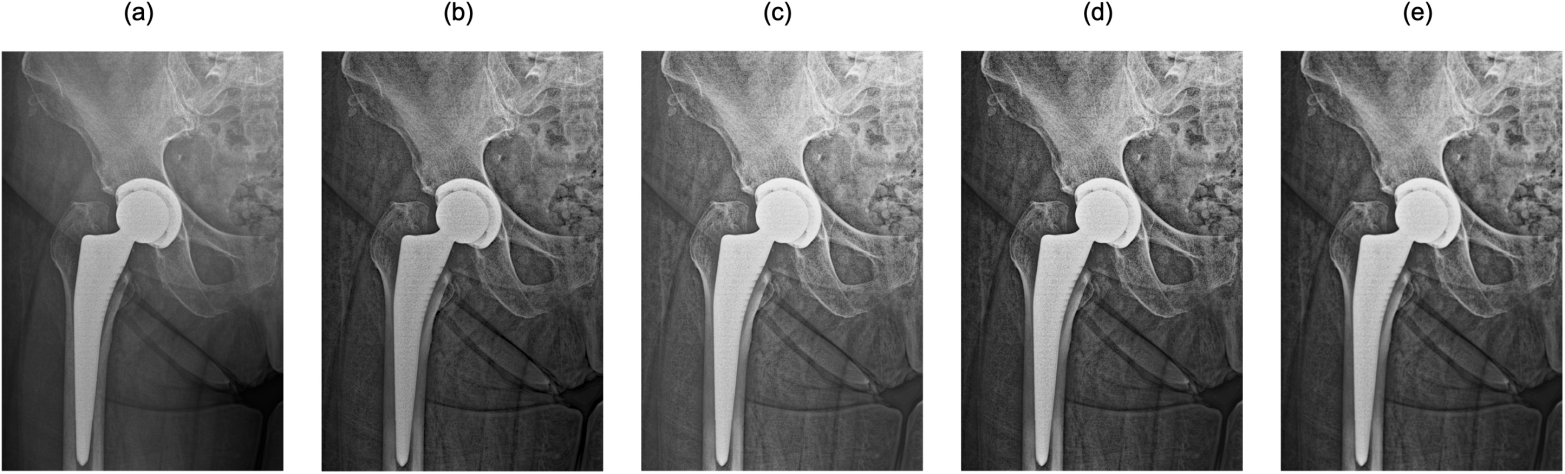
Sequence of preprocessing steps to decrease variability within images. Sequence of: **(a)** Initial image, **(b)** Image after gamma transformation, **(c)** Image after sigmoidal cutoff, **(d)** Image after adaptive histogram equalization, **(e)** Final preprocessed image.

As a preliminary step, radiographs were extracted from the DICOM files and converted to JPEG images using Python’s PyDICOM API. To address the presence of variabilities in the images, e.g., anatomical coverage, image quality, and pixel color range, different preprocessing steps were performed using Matlab2024a, following procedures reported in recent studies [13,21]. First, the Region of Interest (ROI) around the prosthesis was manually cropped from each image, focusing on the acetabulum, femoral head, and periprosthetic bone. Second, images with inverted colors (implant black on a white background) were corrected. Then, a sequence of filtering operations was performed to improve the image quality: (i) gamma power transformation was applied to reduce mist-like effects and enhance brightness; (ii) a sigmoidal function was applied to improve contrast between the background and the prosthesis; (iii) a contrast-limited adaptive histogram equalization (CLAHE) was employed to further highlight the prosthesis compared to the bone structures; (iv) a 2-D Gaussian smoothing kernel was applied as a low-pass filter, to filter out high-frequency noise, enhancing image quality as in [13,21]. Fig 1 shows an example of initial and preprocessed image.

### Model development

**Fig 2:**
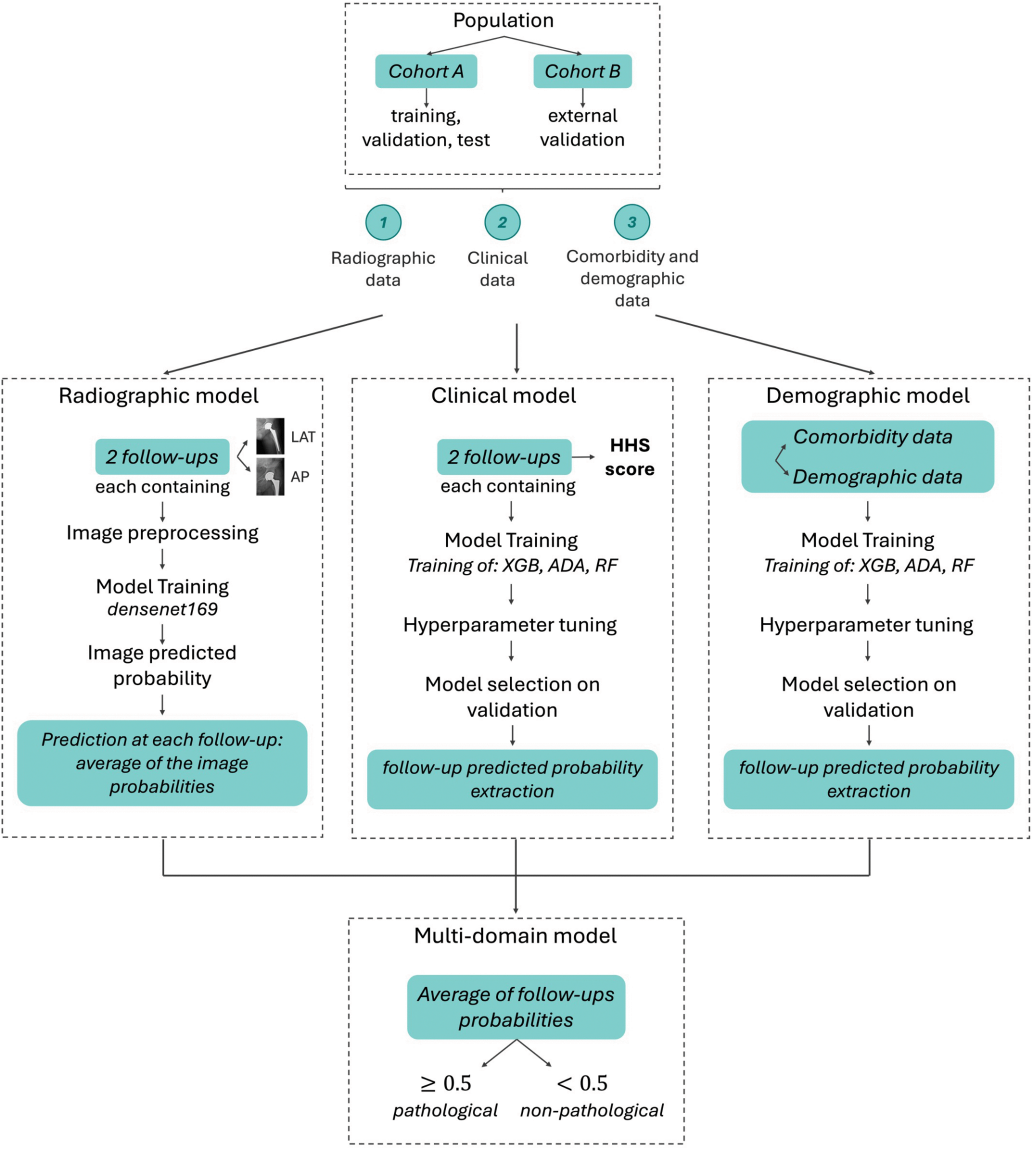
Scheme of the model development workflow.

Three independent models were developed, each using single-domain data, i.e., radiographic, clinical, and demographic. A final multi-domain model was then obtained by integrating the three above-mentioned, leveraging their individual strengths to improve overall performance. The performance of the clinical, demographic, and combined model was evaluated in terms of sensitivity, specificity, F1 score, balanced accuracy, and area under the receiver operating characteristic curve (AUC). An overview of the workflow is shown in Fig 2.

To prevent data leakage across training, validation, and test sets, a patient-level stratified split was applied, ensuring that all follow-up data from the same patient were assigned to the same set, thereby providing an unbiased evaluation of model performance. In addition, label stratification was applied to ensure a balanced distribution of pathological and non-pathological cases across the splits. The final split resulted in 65% of the patients allocated to the training set, 15% to the validation set, and 20% to the internal test set. The number of radiographs in each set is presented in Table 2.

**Table 2:** Patient’s image data set representation and subdivision into training, validation, test, and external validation sets.

	Patients	Images	Pathological images	Non-pathological images
Training	255	1011	511	500
Validation	64	255	120	135
Test	80	320	170	150
External validation	155	925	417	508

#### Radiographic model.

For the radiographic data, a convolutional neural network (CNN) based on a DenseNet-169 backbone pre-trained on ImageNet was employed [22,23]. The network was fine-tuned by unfreezing the last convolutional blocks, while the earlier layers remained frozen to retain general low-level feature representations. A global average pooling layer was added, followed by a dense layer with 128 neurons, batch normalization, and dropout with a 0.3 rate to reduce overfitting. The final classification was performed through a softmax output layer with two units, corresponding to the binary classification task.

During model training, data augmentation was employed to enhance robustness of potential variations in radiographic images and to address the limited size of the dataset. The applied transformations included random rotations of up to ±30°, shearing, scaling (zoom in and out by up to 20%), and horizontal flipping. These augmentations were designed to mimic possible variations encountered in clinical imaging, while preserving anatomically consistent representations.

The model was trained with the Adam optimizer (learning rate = 9 × 10^−5^) and categorical cross-entropy loss. Training was performed with early stopping and learning rate reduction on plateau to prevent overfitting, with performance monitored on the validation set.

After training, the model was evaluated on the test, and external validation sets. For each image, the predicted probability of belonging to the pathological class was extracted. To generate a single prediction associated to each follow-up, the probabilities of all patient images associated with that specific follow-up were averaged. Follow-ups with an average probability ≥0.5 were classified as pathological, while those with an average probability <0.5 were classified as non-pathological. Furthermore, the obtained predicted probability at the follow-up level was subsequently employed in the multi-domain model.

#### Clinical model.

The clinical model was developed based on the total score of the HHS questionnaire recorded at each follow-up. Three machine learning algorithms were applied: AdaBoost (ADA), XGBoost (XGB), and Random Forest (RF). To address class imbalance, Synthetic Minority Oversampling Technique (SMOTE) was applied [24]. Hyperparameters for each model were optimized through a grid search on the training set. The models were first evaluated on the validation to identify the best performing algorithm. The model achieving the highest F1 score, balanced accuracy and AUC was then selected as the final clinical model and subsequently applied to the test and external validation sets. Finally, for each follow-up, the predictive probabilities were extracted and later employed in the multi-domain model.

#### Demographic and comorbidities model.

The demographic and comorbidities model integrated multiple types of data. Among demographic factors, height (cm) and weight (kg) were selected, due to their established influence on THA outcomes [5]. To account for differences in scale and distribution, both variables were standardized using z-score. From the 19 documented comorbidities, only 4 were retained in the final model, to include comorbidities present in at least 10% of cohort A. The retained comorbidities were hypertension**,** cardiac diseases, liver diseases, and thyroid diseases. Moreover, to ensure consistency with the other two models, comorbidity features, which are defined at the patient level rather than at the follow-up level, were replicated across all follow-ups of the same patient and aligned with the corresponding labels.

As with the clinical model, three machine learning algorithms (ADA, XGB, and RF) were applied to the demographic and comorbidity data. To manage class imbalance, SMOTE was applied [24]. The same approach as for the clinical model was used for hyperparameter tuning and evaluation. Following the same workflow, predictive probabilities were extracted at each follow-up and subsequently integrated into the multi-domain model.

#### Combination of the three models.

**Fig 3:**
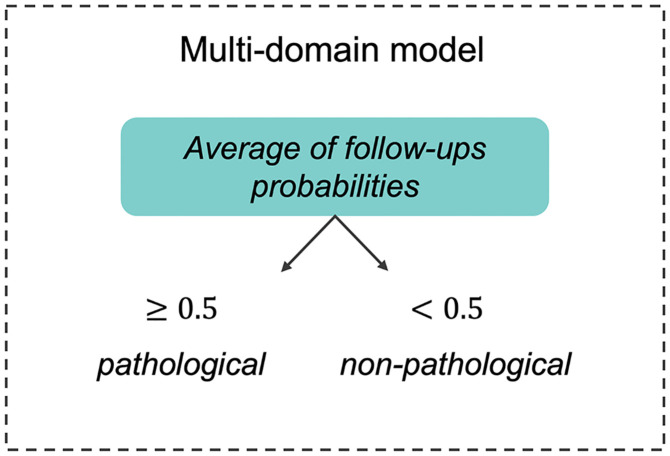
Multi-domain model’s framework.

To assess whether integrating radiographic, clinical and demographic data improves the predictive performance, a combined model was developed. As described above, predictive probabilities were extracted from each model: for the radiographic model at the image level and subsequently averaged to obtain a follow-up level probability, and for the clinical and demographic models directly at the follow-up level. The combined probability for each follow-up was then obtained as the average of the three model probabilities. Then, as for the radiographic model, the final prediction for each follow-up was obtained by using the same decision rule: follow-up with an average probability ≥0.5 were classified as pathological and those at or below 0.5 as non-pathological (Fig 3). Metrics are reported as the mean across 100 bootstrap resamples, with corresponding 95% confidence intervals.

## Results

Considering results on the validation set, the RF achieved the best performances in terms of balanced accuracy, F1 score and AUC, both in the clinical and demographic models, thus it was selected for the next steps in the combined multi-domain approach. S1 and S2 Tables summarize the performance metrics of the clinical and demographic models on the validation set, respectively.

Table 3 summarizes the performance metrics on the internal test, and external validation sets for each of the three mono-domain models, as well as for their combined approach. Metrics are reported as the mean across 100 bootstrap resamples, with corresponding 95% confidence intervals.

**Table 3:** Performance metrics of the combined model on the test and external validation.

Internal Test					
	F1 score	B Acc	Specificity	Recall	AUC
*Radiographies*	0.68 [0.61, 0.75]	0.64 [0.57, 0.70]	0.55 [0.44, 0.64]	0.73 [0.65, 0.81]	0.72 [0.64, 0.78]
*Clinical*	0.61 [0.51, 0.68]	0.58 [0.49, 0.66]	0.55 [0.43, 0.66]	0.61 [0.49, 0.70]	0.60 [0.50, 0.69]
*Demographic*	0.62 [0.54, 0.69]	0.64 [0.58, 0.70]	0.71 [0.60, 0.82]	0.57 [0.54, 0.65]	0.63 [0.54, 0.70]
*Combination*	0.72 [0.65, 0.79]	0.69 [0.63, 0.77]	0.64 [0.52, 0.75]	0.74 [0.67, 0.82]	0.72 [0.65, 0.79]
**External Validation**					
	**F1 score**	**B Acc**	**Specificity**	**Recall**	**AUC**
*Radiographies*	0.62 [0.59, 0.66]	0.59 [0.52, 0.62]	0.37 [0.33, 0.43]	0.81 [0.76, 0.85]	0.64 [0.58, 0.69]
*Clinical*	0.65 [0.61, 0.69]	0.61 [0.58, 0.63]	0.36 [0.31, 0.44]	0.86 [0.81, 0.90]	0.63 [0.59, 0.67]
*Demographic*	0.48 [0.43, 0.54]	0.52 [0.48, 0.56]	0.53 [0.48, 0.59]	0.50 [0.44, 0.58]	0.54 [0.49, 0.59]
*Combination*	0.66 [0.62, 0.70]	0.62 [0.59, 0.66]	0.40 [0.34, 0.45]	0.85 [0.80, 0.89]	0.67 [0.62, 0.72]

Metrics are reported as the mean [95% CI] across 100 bootstrap resamples, with corresponding 95% confidence intervals. B Acc: balanced accuracy; AUC: Area under the receiver operating characteristic curve.

**Fig 4:**
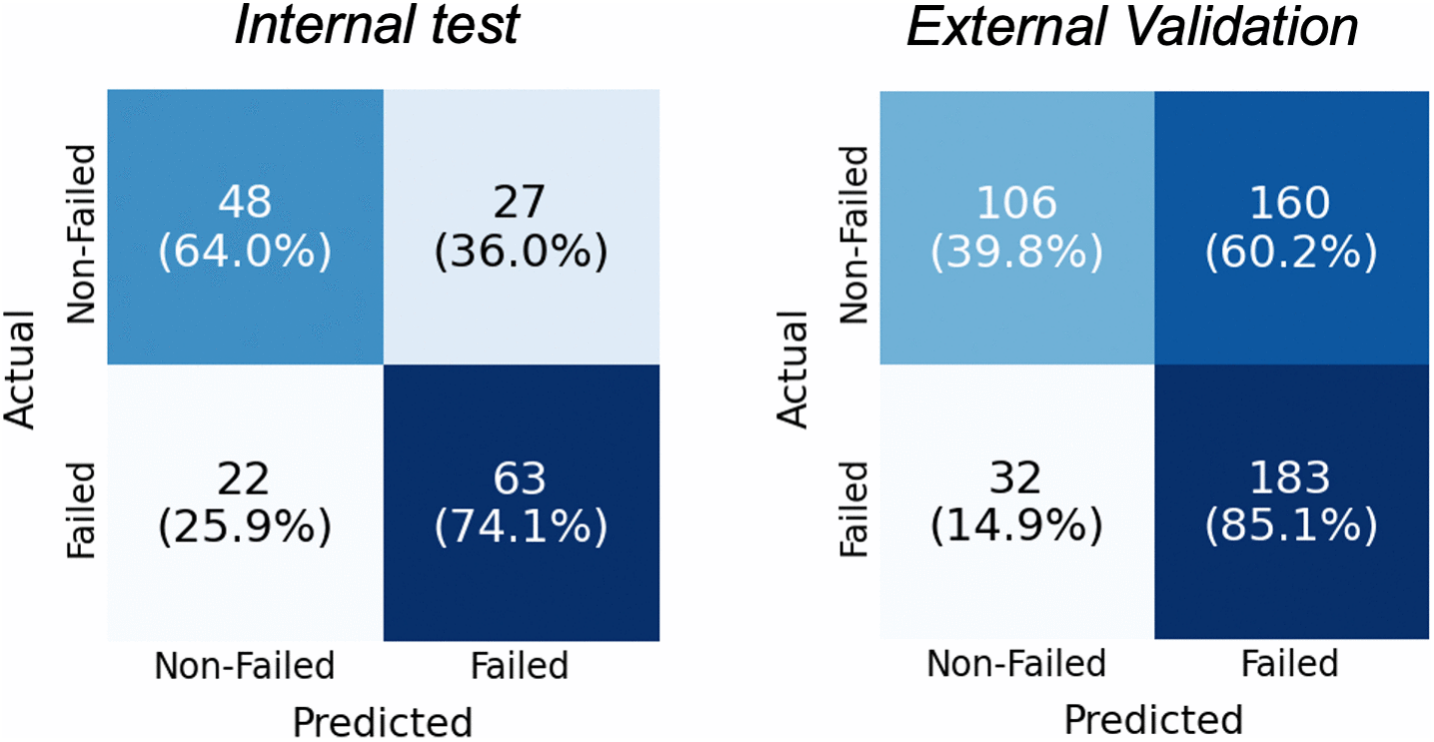
Confusion matrices of the follow-ups obtained across the different subsets of data, including internal test and external validation sets.

Fig 4 illustrates the confusion matrices for the internal test, and external validation sets obtained with the combined model, where each instance corresponds to a patient’s follow-up.

On the internal test set, the combined model produced 48 true negatives and 27 false positives, corresponding to 64.0% and 36.0% of the actual non-failed cases, respectively. Among the actual failed cases, it yielded 63 true positives and 22 false negatives, corresponding to 74.1% and 25.9% of the actual failed cases, respectively. The corresponding misclassification error rate was 30.6%. These results corresponded to a positive predictive value (PPV) of 0.70 and a negative predictive value (NPV) of 0.69, indicating that most errors were driven equally by false positive and false negative classifications.

On external validation, the combined model yielded 106 true negatives (39.8%), 160 false positives (60.2%), 32 false negatives (14.9%), and 183 true positives (85.1%). The corresponding misclassification error rate was 39.9%, indicating that most errors were driven by false positive classifications. In this cohort, the model achieved a PPV of 0.70 and a NPV of 0.77.

**Fig 5:**
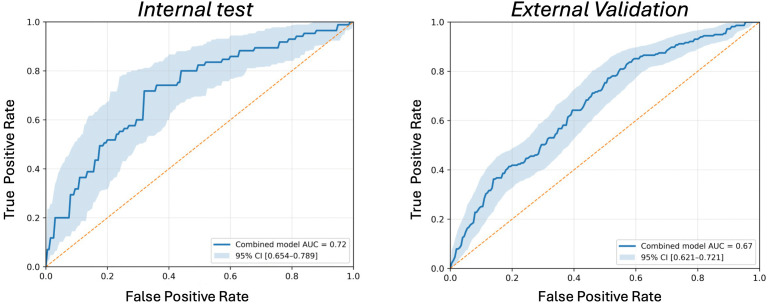
Receiver operating characteristic (ROC) curves for the combined model on the internal test set (left) and external validation cohort (right).

Additionally, the ROC curves of the combined model for the internal test set and the external validation cohort are reported in Fig 5. The combined model achieved an AUC of 0.72 (95% CI: 0.654–0.789) on the internal test set and 0.67 (95% CI: 0.621–0.721) on external validation, indicating a decrease in performance when evaluated on the external validation.

Calibration curves for the combined model on both the internal test set and the external validation cohort are reported in S1 Fig, showing reasonable agreement between predicted probabilities and observed outcomes, although a tendency toward probability overestimation was observed in the external validation cohort.

To further assess the potential clinical utility of the proposed combined model, a decision curve analysis (DCA) was performed and reported in the Supplementary Material (S2 Fig). For the internal test set, the combined model provided a higher net benefit than both treat-all and treat-none strategies for threshold probabilities between 0.30 and 0.70, indicating potential clinical usefulness across a broad range of decision thresholds. Similarly, on the external validation cohort, the model demonstrated a positive net benefit for thresholds between 0.30 and 0.55.

## Discussion

This study emphasizes the value of a multi-domain analysis that combines post-operative radiographs with clinical, demographic and comorbidity data to improve the early detection of pathological signs after primary THA. The main findings of this study are: i) the implementation of a multi-domain approach that integrates imaging with clinical and comorbidity information relevant to implant function; ii) the application of this approach to radiographs acquired within the first 12 months after primary THA, rather than pre-revision images, which enables early identification of pathological signs; and iii) the demonstration that such an approach can be used as a virtual follow-up visit, potentially reducing the need for in-person evaluation and the burden on clinicians.

Unlike previous studies that primarily used pre-revision images [5,12–16, 25] our model was applied to post-operative follow-ups, thus enabling early detection of post-operative pathological signs. It is important to underline, however, that the presence of such early signs does not necessarily imply implant failure or the need for revision surgery. This makes the predictive task less evident and more complex than classifying implants that have already failed, as done in previous studies. Nevertheless, this focus provides greater clinical value, as it aims to identify early pathological signs that can guide closer monitoring and timely intervention. Moreover, the proposed approach, by uniquely integrating radiographic, clinical, comorbidities, and demographic data, including HHS scores, provides a more comprehensive evaluation that combines imaging with symptomatic information relevant to implant function. For instance, Loppini et al. [13] developed a DL model to detect THA loosening from preoperative radiographs, reporting an accuracy of 97% on a relatively small test set of 185 images. Similarly, Borjali et al. [25] applied a DL model to preoperative radiographs of 236 patients, achieving 91.6% of sensitivity and 75% specificity in detecting THA loosening. In another study, Borjali et al. [15] developed a convolutional neural network to identify implant designs from AP radiographs, reaching an accuracy of 100% on a test set of only 25 patients. Muscato et al. [14] proposed a hybrid DL–ML model using exclusively radiographic data before revision surgery, achieving 95.8% accuracy and an F1 score of 0.874. The higher performance associated with these studies compared to our findings may reflect the greater difficulty of identifying early pathological signs within the first year after surgery rather than predicting implant failure immediately before revision surgery. Additionally, Bulloni et al. [16] achieved an AUC of 0.95 through a repeated nested cross-validation, by relying solely on clinical data in a cohort of 162 patients. Finally, a significant innovation was introduced by Shah et al. [12] who developed a DL model combining radiographs with clinical history in a cohort of 697 patients, demonstrating that integrating clinical data improved performance and resulted in 97.5% accuracy in detecting implant loosening. Our findings are consistent with this evidence, as the addition of HHS scores and comorbidities improved prediction compared to the radiographs alone.

This study presents a novel approach for detecting pathological signs within one year after primary THA using radiographic, clinical, and comorbidity data, thus enabling a virtual follow-up. Moreover, combining the three models by averaging their probabilities improved the balance between sensitivity and specificity and enhanced overall model generalizability.

However, the study has certain limitations that should be considered when interpreting the results. While the model demonstrated high sensitivity on both the internal test set (0.74) and external validation cohort (0.85), effectively detecting most early pathological signs, the specificity was lower, particularly on the external validation (0.40). This pattern suggests that the model tends to classify a relatively broad set of follow-ups as potentially pathological, capturing most true positive cases while also including a proportion of non-pathological ones. In this setting, predictive values offer a more clinically meaningful interpretation of model output. The PPV of 0.70 indicates that nearly 7 out of 10 follow-ups classified as pathological by the model corresponded to true pathological findings. Likewise, the NPV, ranging from 0.69 to 0.77, suggests that about 7–8 out of 10 follow-ups classified as non-pathological were correctly identified. Moreover, the combined model yielded a misclassification error rate of 30.6% on the internal test set and 39.9% on external validation, indicating that approximately 3 out of 10 and 4 out of 10 follow-ups, respectively, were incorrectly classified. This confirms that, although the model was able to detect most pathological cases, classification errors remained substantial, particularly in the external validation cohort.

Nevertheless, although generally less clinically critical than false negatives, false positive predictions should not be considered entirely benign. In clinical practice, they may lead to additional imaging examinations, increased healthcare utilization, greater clinician workload, and potential patient anxiety. Therefore, the clinical implications of reduced specificity should be carefully evaluated when considering the integration of such models into follow-up workflows. In addition, these findings may suggest that the deep learning model was able to identify subtle pathological features that were not yet clearly recognizable during clinical assessment, highlighting its potential role in supporting the early identification of pathological signs on radiographs.

To better assess the potential clinical impact of the proposed approach, calibration and DCA were also performed. Calibration curves showed a reasonable agreement between predicted probabilities and observed outcomes, although a tendency toward probability overestimation was observed in the external validation cohort. This indicates that the model tends to assign slightly higher risk estimates than those observed in practice, suggesting a degree of overconfidence when applied to independent data. In practical terms, this behavior may contribute to the increased number of false positive predictions observed in the external validation set, as some follow-ups are assigned higher predicted probabilities of pathological findings than those actually occurring. However, this behavior is also consistent with the sensitivity performance profile of the model and reflects the intrinsic difficulty of detecting subtle pathological signs during the early post-operative period. In addition, DCA demonstrated that the combined model provided a positive net benefit compared with both treat-all and treat-none strategies across a range of clinically plausible threshold probabilities, including the 0.5 threshold adopted in the present study. However, as no threshold optimization was performed, this finding should not be interpreted as evidence that 0.5 represents the optimal operating threshold for clinical implementation. Rather, it suggests that the model may have potential utility, while the most appropriate threshold should be defined according to the intended clinical application and the relative consequences of false positive and false-negative predictions. These findings suggest that, despite the lower specificity, the model may still support follow-up decision-making by identifying patients who may benefit from closer monitoring. However, the potential increase in clinical workload associated with false positive predictions should be taken into account when considering its practical implementation.

Overall, the moderate performance highlights that identifying early signs of failure remains a challenging task. This is likely due to the inherent difficulty of detecting pathological signs immediately post-operatively, when radiographic changes may be subtle or not yet apparent, and the eventual outcome of the implant is unknown. The lower performance observed on the external validation set highlights the need for future studies to expand the dataset or apply the model to another independent patient cohort to further increase the model’s robustness. Additionally, incorporating further clinical and demographic variables, along with features extracted directly from radiographs, may improve predictive accuracy.

## Conclusion

This study introduces the first integrated DL and ML framework for detecting pathological signs within one year after primary THA, leveraging radiographic, clinical, demographic, and comorbidity data to enable a virtual follow-up. The combination of the three models through probability averaging enhanced the overall predictive performance, compared to single models. Moreover, the external validation supported its potential clinical applicability. Despite some limitations in specificity, the model may stll support follow-up decision-making by identifying patients who could benefit from closer monitoring, although the potential impact of false positives on clinical workload should be considered. Nonetheless, further validation on larger, multicentric cohorts will be essential to confirm its robustness and ensure broader clinical adoption.

## Supporting information

S1 Table Performance metrics of the clinical model on the validation set. (DOCX)

S2 Table Performance metrics of the demographic model on the validation set. (DOCX)

S1 Fig Calibration assessment curves of the combined model on the internal test set and external validation set. (TIF)

S2 Fig Decision curve analysis of the combined model on the internal test set and external validation set. (TIF)
