# Supplementary material for: Automatic early detection of pathological signs following primary total hip arthroplasty using radiographs, clinical scores, and comorbidities
Source: PLoS One. 2026 Jun 15;21(6):e0348790. doi: 10.1371/journal.pone.0348790 (PMC13268146; doi:10.1371/journal.pone.0348790)
Supplement: S1 Table — (DOCX) [file pone.0348790.s001.docx]

**S1 Table. Performance metrics of the clinical model on the validation set.**

| **Clinical model** | | | | | |
| --- | --- | --- | --- | --- | --- |
|  | **F1 score** | **B Acc** | **Specificity** | **Recall** | **AUC** |
| *RF* | 0.66  [0.56, 0.74] | 0.65  [0.58, 0.73] | 0.58  [0.47, 0.71] | 0.72  [0.60, 0.82] | 0.66  [0.57, 0.73] |
| *XGB* | 0.65  [0.55, 0.72] | 0.61  [0.54, 0.68] | 0.45  [0.33, 0.56] | 0.77  [0.65, 0.86] | 0.62  [0.53, 0.70] |
| *ADA* | 0.64  [0.55, 0.72] | 0.60  [0.54, 0.67] | 0.44  [0.32, 0.55] | 0.77  [0.65, 0.86] | 0.61  [0.52, 0.69] |

Results are reported as the mean [95% CI] across 100 bootstrap resamples. B Acc: balanced accuracy; AUC: Area under the receiver operating characteristic curve; RF: Random Forest; XGB: XGradient Boosting; ADA: AdaBoost.
