# Supplementary material for: Automatic early detection of pathological signs following primary total hip arthroplasty using radiographs, clinical scores, and comorbidities
Source: PLoS One. 2026 Jun 15;21(6):e0348790. doi: 10.1371/journal.pone.0348790 (PMC13268146; doi:10.1371/journal.pone.0348790)
Supplement: S2 Table — (DOCX) [file pone.0348790.s002.docx]

**S2** **Table. Performance metrics of the demographic model on the validation set.**

| **Demographic model** | | | | | |
| --- | --- | --- | --- | --- | --- |
|  | **F1 score** | **B Acc** | **Specificity** | **Recall** | **AUC** |
| *RF* | 0.60  [0.50, 0.69] | 0.60  [0.50, 0.67] | 0.55  [0.43, 0.66] | 0.64  [0.50, 0.75] | 0.59  [0.47, 0.70] |
| *XGB* | 0.58  [0.46, 0.69] | 0.55  [0.46, 0.63] | 0.45  [0.32, 0.55] | 0.66  [0.54, 0.79] | 0.58  [0.47, 0.69] |
| *ADA* | 0.46  [0.36, 0.56] | 0.50  [0.40, 0.58] | 0.55  [0.45, 0.66] | 0.45  [0.33, 0.57] | 0.54  [0.45, 0.64] |

Results are reported as the mean [95% CI] across 100 bootstrap resamples. B Acc: balanced accuracy; AUC: Area under the receiver operating characteristic curve; RF: Random Forest; XGB: XGradient Boosting; ADA: AdaBoost.
